# Potential trajectories of the upcoming forest trading mechanism in Pará State, Brazilian Amazon

**DOI:** 10.1371/journal.pone.0174154

**Published:** 2017-04-05

**Authors:** Brenda Brito

**Affiliations:** 1 Stanford Law School, Stanford University, Palo Alto, California, United States of America; 2 Amazon Institute of People and the Environment (Imazon), Belém, Pará, Brazil; University of Vermont, UNITED STATES

## Abstract

In 2012, the Brazilian government revised the federal Forest Code that governs the use of forest resources on rural properties. The revisions included a forest trading mechanism whereby landowners who deforested more than what is legally allowed before 2008 could absolve their deforestation “debts” by purchasing Environmental Reserve Quotas (CRA) from landowners who conserved more forest than legally required. CRA holds promise as a tool to complement command-and-control initiatives to reduce deforestation and incentivize restoration. However, the success of this instrument depends on how its implementation is governed. This study builds on a few recent assessments of the potential of the CRA in Brazil–but that are focused on biophysical potential–by assessing how a few key implementation decisions may influence the CRA market development. Specifically, this study estimates how decisions on who can participate will likely influence the potential forest surplus and forest debt for the CRA market, and takes into account governance characteristics relevant to the State of Pará, eastern Amazonia. In particular, the study evaluates the effects in the CRA market eligibility after simulating a validation of properties in the environmental rural registry (CAR) and assessing different scenarios surrounding land tenure status of properties. Results show how regulatory decisions on CRA market eligibility will determine the extent to which CRA will serve as a tool to support forest conservation or as a low-cost path to help illegal deforesters to comply with legislation, but with limited additional environmental benefits. The study reviews regulatory options that would reduce the risk of forest oversupply, and thereby increase the additionality of the areas eligible for CRA. Overall, the study demonstrates the importance of including governance as well as biophysical characteristics in assessing the potential of forest trading tools to deliver additional environmental conservation and restoration benefits.

## Introduction

Brazil has been widely recognized for reducing deforestation in the Amazon forest between 2005 to 2014. Researchers have linked the decline in deforestation to both innovative command and control policies, which included more strategic enforcement actions, as well as to measures adopted by actors in major agricultural commodities, such as cattle and soy beans, towards a more sustainable production [1–4]. However, these types of policies may not be sufficient to sustain low-deforestation rates in the long term, or to reach zero deforestation. Thus, it is of interest to explore how other instruments that provide positive incentives to reduce deforestation may play a complementary role [2].

One of such incentives recognized by current Brazilian law is the Environmental Reserve Quota (henceforth CRA, for its Portuguese acronym). CRA allows private landowners who deforested beyond the permissible amount up to 2008, to offset their forest pre-2008 “debts” by paying someone else who conserved more forest than was legally required.

The CRA, in its essence, fits the definition of Payment for Ecosystem Services program [5]: it is a voluntary transaction where a well-defined environmental service (here, services associated with forest ecosystems) is being ‘bought’ by a service buyer (here, the property owner who lacks forest area in his legal reserve) from a service provider (here, the property owner with the additional forest) if the service provider secures service provision (here, the landowner agrees to conserved the area for the duration of the contract).

While all rural property owners in Brazil are required to maintain a minimum percentage of forested area (called legal reserve), CRA can be considered a “carrot” to help them comply with this obligation at a lower cost, while also rewarding those who conserved more forest areas than required. In this way, CRA can be considered as a mix between command and control and incentive mechanisms [6] that provides flexibility to land owners in complying with the new Forest Code. It also operates like a development rights transfer, since the landowners with illegally deforested area can continue to exploit (or develop) their lands in exchange for a payment to set aside another location [7].

However, the CRA market also has stringent rules on participation: on the demand side, CRA may only compensate areas illegally deforested in legal reserves before July 28, 2008, and the supplied “offset” area for compensation must be in the same biome as the demand area. If deforestation beyond the permitted amount occurred after July 28, 2008, the property owner must restore that area. At the same time, a noncompliant property owner eligible for CRA can opt to restore their forest debt areas instead of participate in the CRA market.

At the time of this publication, studies evaluating the potential of the CRA in the Brazilian Amazon have largely focused on biophysical determinants of CRA potential supply and demand, estimating the market potential by comparing the amount of available forest resources eligible for the CRA market (forest surplus) and areas of pre-2008 non Forest-Code compliance (forest debt). In the first study of CRA at national scale, Soares-Filho and colleagues [8] estimated that CRA could compensate 56% of the environmental liabilities in Legal Reserves in Brazil. The authors also found that three states of the Amazon region (Acre, Pará and Rondônia) could compensate all pre-2008 environmental debt through CRAs. In a study of the Amazonian State of Mato Grosso, the authors estimated that the forest surplus for CRA would exceed the forest debt [9]. Yet, another recent analysis, but focused on the state of Pará, estimated that forest supply would exceed the forest pre-2008 debt by more than a factor of five [10].

These studies took into account primarily the existence of forest resources and the cleared area for estimating forest surplus and debt for CRA. However, biophysical factors are not the sole determinants of CRA’s potential: legal and institutional factors will also determine what properties are eligible to participate in this mechanism. Studies that neglect these institutional factors may thus overestimate the real potential of the CRA market. While two studies sought to use data on land tenure status to estimate potential supply of CRA in Brazil [11,12], they may also have overestimated the market, as there is a chance that areas pending a land title were considered as regularly titled in their assessments. This matters in the Brazilian Amazon in particular, as land tenure conflicts abound, and because non-titled lands cannot use their forest surplus in the CRA market.

Thus, as a contribution to this recent literature, this study aims to provide a more in-depth assessment of how the governance factors may shape the CRA market development in Pará. Pará is of interest as it ranks among the Amazonian states with the highest rates of deforestation, but the state also has a large remaining forested area (around 80% of its territory), making CRA a potentially valuable instrument for Pará in particular.

This study has two main hypotheses: i) adding governance requirements will reduce the forest surplus estimates, compared with previous studies and ii) the revised CRA supply will be so low that it will not be able to compensate for all pre-2008 forest debts in Pará.

To address these hypotheses, this assessment focuses on three main questions. First, what are the main legal and institutional requirements for CRA implementation? Second, taking into account such requirements, what is the estimated forest surplus and debt eligible for CRA transactions in Pará? Third, after taking into account these governance requirements, is Pará likely to run the risk of excess forest supply—as suggested by previous literature [9–12]–, and, if so, what are possible alternatives to reduce this risk?

The research draws upon interviews with key stakeholders and legal content to determine what major legal and institutional factors must exist in order for the CRA market to function. Then, based on the findings, I developed three scenarios of forest surplus and pre-2008 debt for Pará CRA market, as well as a five-year projection of expected eligible forest surplus for CRA in Pará. These analyses help address under which conditions CRA oversupply is likely. CRA oversupply matters, as it would likely result in a low CRA price. A low CRA price, in turn, could limit the appetite of property owners to supply their forest surplus areas to the market, especially those in areas of high deforestation pressure. It might also reduce the incentive to pursue in-situ conservation for those with pre-2008 forest deficits. Overall, oversupply conditions will likely reduce CRA’s potential forest conservation and restoration benefits.

Based on the findings and on the existing literature, the article concludes by reviewing the policy options that may determine whether the main contribution of CRA in the next years is as a market-based mechanism for legalizing illegally deforested areas, or as a forest conservation mechanism that can support the provision of additional environmental services.

## Environmental reserve quotas—CRA

### Legal reserve and the CRA

**Fig 1:**
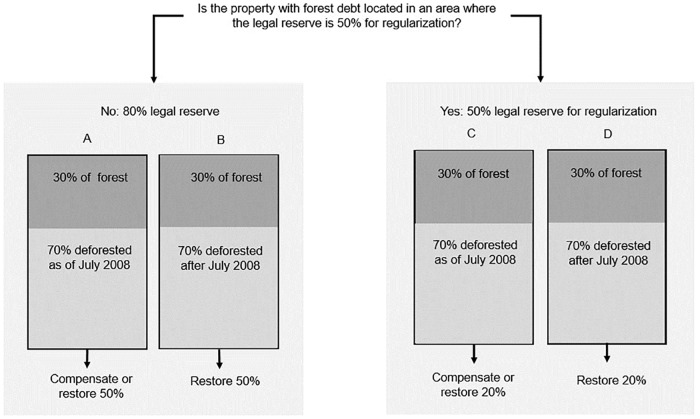
Examples of the legal reserve rules for properties deforested before and after July 2008. This example applies for medium and larger properties, since small properties (up to 64 hectares in average in Pará State) do not need to restore or compensate legal reserve deforested as of 2008. Property A and C have a pre-2008 legal reserve debt, but only property A is located in a region where the legal reserve can be reduced to 50% for regularization purposes. The landowner of property A needs to regularize 50% of the property with compensation or restoration to reach a 80% legal reserve, while property C needs a 20% regularization to reach a 50% legal reserve. Properties B and D have a post-2008 legal reserve debt, but in both cases their landowners can only restore the deforested area.

The Brazilian Forest Code requires that all rural properties maintain a minimum amount of forest areas, called the legal reserve. The size of the legal reserve depends on the biome in which the property is located. In the Amazon biome, the general rule is a legal reserve of 80% of the property’s area. However, the new Forest Code (Federal Law number 12,651 from 2012) introduced some exceptions for some deforested legal reserves depending on the property size and location. Fig 1 presents the most common exceptions to the 80% rule for medium and large properties in Pará. See S1 File for more details on legal reserve size and exceptions.

Of particular note, the new Forest Code allows rural property owners who did not maintain their legal reserve up through July 2008 (referred in this study as forest debt) to absolve their debts by financing forest areas in other properties that have more forest than legally required (referred in this paper as forest surplus). The Environmental Reserve Quota (CRA) program facilitates the exchange among lands in the same biome, where each CRA certificate corresponds to one hectare of either intact forest or forest in the process of regeneration or restoration. The environmental agency must verify the technical feasibility of regeneration or restoration before issuing the CRA.

While the CRA supplier and demander must be in the same biome, some biomes cross state lines, and each state must decide whether they permit trading across state-lines in their same biome. For instance, consider the case of a Pará property owner with a forest debt who wishes to compensate in a property from Amazonas State to comply with the Forest Code. Current Pará legislation will only permit this trade if the property owner with the forest debts proves that it is economically and technically inviable to compensate a suitable forest area in Pará. In addition, the Forest Code has allowed interstate compensation to occur when the area to be compensated is in “priority areas”. Either the Federal government or the Pará government could designate what is a priority area, however, neither have established those as of 2016. Overall, until this classification occurs, the CRA market in Pará will likely be restricted to properties within the state.

The amount of potentially available forest on a given property eligible for the CRA market depends on the property’s size and location. The simplest case is that of small properties (on average less than 64 hectares for Pará): their landowners can use any remaining forest area on those properties to supply the CRA market. Medium and large-properties can use forest areas beyond 50 or 80% of their property for CRA certificates, depending on their location (Fig 1). However, forest areas beyond 80% of the property could, alternatively, be legally deforested, regardless of their size and location. This means that property holders face a tension between allocating forested areas exceeding 80% of the property to either the CRA market or to implement other land use types, such as agriculture. S1 File details the rules applicable for determining the amount of forest surplus in a given property.

### Registration of CRAs

In order to issue or use the CRA, a given property must be recorded in the Rural Environmental Registry (henceforth CAR, for its Portuguese acronym). The CAR is a mandatory registry for all rural properties in Brazil (estimated at 5 million), and it requires the both georeferenced data on property’s borders and information about the property’s compliance with the forest code, which includes any unauthorized deforestation in the legal reserve area. Once in this registry, the state environmental agency must verify and validate the property information before the CAR is approved. For instance, if different properties overlap in anyway, CAR registrations will be considered pending and eventually cancelled if the overlap is not solved. Parcels that do not comply with environmental regulation, e.g. deforestation above the legally permitted amounts, will need to regularize their situation

**Fig 2:**
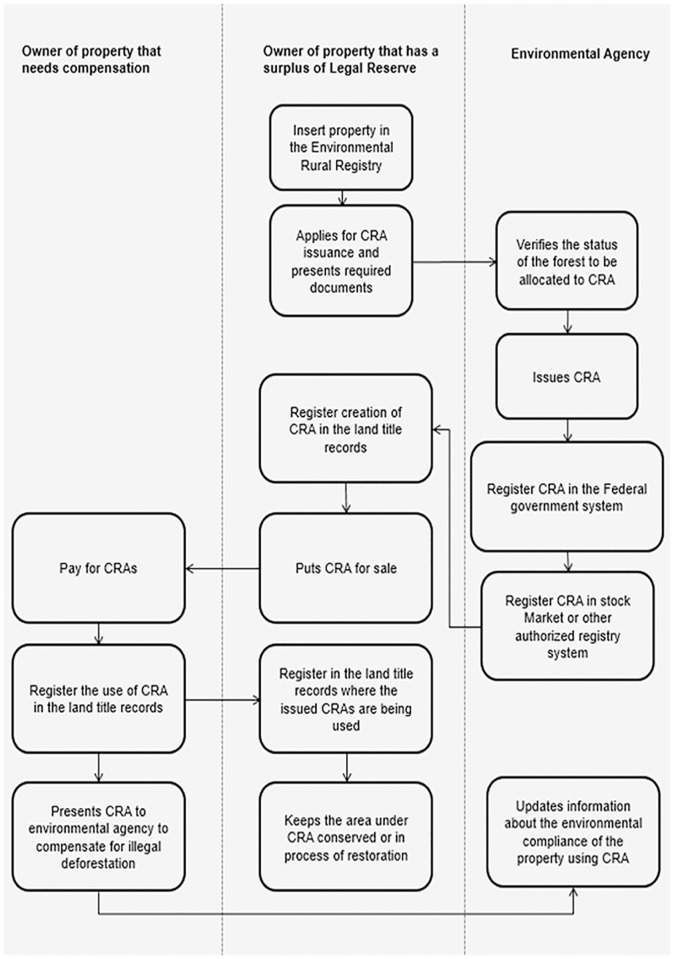
Steps for issuing and using CRAs to compensate illegal deforestation up to 2008.

Issuing a CRA certificate requires several steps. First, if the property has more forest than required by the Forest Code (a property with a forest surplus), the landowner must request that the state environmental agency issue the CRAs during registration in the environmental rural registry (CAR). The state agency must then register each CRA in a central system controlled by the federal government (called SICAR) and each CRA will receive a unique identification number. Once the CRA has its ID, the environmental agency has 30 days to register the CRAs with stock markets or registry systems authorized by the Central Bank of Brazil. Currently only two institutions in Brazil have such authorization: the São Paulo Stock Market (BM&F Bovespa) and Cetip. Once the CRA is registered in the stock market, the CRA owner can then transfer it to third parties and inform the environmental agency about the transaction. The agency then updates this information in SICAR. Fig 2 presents all these steps for the issuance and use of the CRA.

Even though the regulation that permit the use of the CRAs has not been finalized as of 2016, some institutions are already organizing databases of potential buyers and sellers of CRAs. The largest one in Brazil is managed by BVRio, an environmental services stock market that created BVTrade, a business platform for several environmental services, including the CRAs. As of November 2014, there were fifty properties in the Amazon region offering CRAs in BVTrade, of which twelve were in Pará State[13].

### CRA’s characteristics and the role of land tenure

CRA can be considered as Payment for Environmental Service in which the government works as a mediator [6], since the environmental agency needs to validate the CRA before the property owners with the forest debts can purchase the CRA and offset their obligation. As a result, the environmental services provider (here, the owners of properties with forest surpluses) and users (here, the landowners with forest debts) can enter and exit contracts voluntarily [14]. Table 1 provides an overview of the critical aspects of the CRAs and how they are transacted.

**Table 1:** Characteristics of the CRA based on [14].

Main parties	1) Property owner with forest surplus and 2) person in charge of a property with legal reserve debt as of July 28, 2008. Both properties must be in the same biome and, if determined by state regulation, in the same state.
Intermediaries	Environmental agency at state or municipal level
Monitoring	By the environmental agency; parties may also agree on additional monitoring, e.g. annual report about the status of the forest surplus)
Conditionality for payment	Conservation of forest area or progress in the restoration/regeneration process
Link to other policies	Conservation of Legal Reserve area according to the Forest Code
Mode of payment	Determined by transaction parties, no restrictions
Timing of payment	Lump-sum payment at beginning of contract or annual payments, depending on what parties agree upon
Contract duration	According to what parties agree upon

Land tenure status plays an important role in determining a property’s eligibility to participate in the CRA market. The forest code is ambiguous as to whether properties lacking land title (henceforth referred to as parcels) can issue a CRA or even use it to compensate for legal reserve debts. A draft Federal Decree from December 2014 about regulating the CRAs market indicated that only properties with land titles could issue a CRA, but that all types of properties, included the non-titled could use CRA’s compensate for forest debts. In Pará, a State Decree from 2015 allows non-titled parcels to use other types of compensation instruments similar to the CRA to offset their debts, e.g. transactions using civil law contracts to rent a forest surplus area from a titled property and compensate for a forest debt in an non-titled parcel. However, the same State Decree does not explicitly mentions this possibility to the CRA, leaving the decision up to federal regulation.

Thus, depending on the final text of this Federal Decree, amount of eligible forest surplus and debt for the CRA market in the Amazon region may vary widely, since many parcels in the Brazilian Amazon do not have title to the property. While no official number exists that quantifies how many properties lack title, Barreto and colleagues estimated that only 4% of the Amazon region is made up of private properties with valid land title as of 2008 [15]. In addition, according to the federal government, at least 160,000 properties in the Amazon region were in “pending” land title as of 2009 [16].

### The risk of forest surplus excess

The decision as to which properties are eligible to issue CRAs will directly affect the amount of forest surplus available for the CRA market. Previous studies have raised concerns about potential excess of forest surplus compared to forest debt [9,11,12], since the potential higher supply compared with demand would result in a lower CRA price. A low CRA price, in turn, could have three main consequences:

1. Limit incentive for conserving high-risk deforestation areas. When property owners with forest surplus areas have alternatives that compensate them better than the CRA, they are more likely to pursue those alternatives than to offer their excess forest to the CRA market. In particular, low CRA prices could fail in attracting property owners of areas with high pressure for deforestation, because of their high opportunity costs. In other words, property holders with higher opportunity costs would be more likely to deforest their forest surplus areas than to use the CRA to support conservation. In such a situation, low payments would decrease the chance of adopting socially-desirable land use practices (in this case, conservation) because the CRA would tend to attract participants whose opportunity cost of participation is low or negative [17].
2. Increase rate of rewarding non-additional behavior. By a similar logic to the previous, low CRA prices would also attract many landowners whose opportunity costs of participation are lower than the CRA payments. Such property owners are more likely to conserve the forest surplus anyway. In this case, CRA would end up rewarding non-additional behavior [6].
3. Reduce incentive for in-situ restoration. The CRA would support in-situ restoration in cases where the costs of participating in the CRA is higher for regularizing the legal reserve. However, the excess of CRAs at a low cost would incentivize all properties with pre-2008 forest debt to use CRA instead of restoration.

Taken together, the excess of forest surplus would affect the environmental integrity of the market, as low prices would not only reduce the incentive to restore on-site, but also reduce the chance of conservation in high deforestation risk areas. In other words, a potential oversupply in the CRA market could make the CRA a low cost way to legalize the situation of illegally deforested properties and reduce the chance of using this instrument to foster conservation and restoration of strategic and sensitive areas.

In order to avoid such negative effects as those mentioned above, one tool that could enhance the environmental benefits of CRA would be additional market regulations that prioritize, or target, environmentally sensitive areas for participation. For instance, as suggested by Wünscher and colleagues [18] for regions where deforestation threats vary across the landscape, a possible targeting criterion would be deforestation rate. Thus, only the forest surplus in areas with high deforestation rates would be eligible for the CRA market. A second option for controlling the size of available forest surplus could involve the government as a direct participant in the market, buying up lower cost CRAs so as to maintain market prices that attract property holders in higher-risk areas to participate. This type of governmental intervention has been used in the US with transferable development rights (TDRs). TDRs are an instrument used in US real estate markets that have similar characteristics to CRAs, in that it is a voluntary, incentive-based program in which one landowner may sell their development rights to another interested party, who can then use the purchased rights to modify their land use practices at another location [19]. A third way to minimize forest oversupply would be to introduce price differentiation, so as to compensate areas with higher deforestation pressure more than areas with lower pressure. Organizing auctions is one way of reaching such differentiation [20]. More specifically, it is possible to use reverse auctions in which landowners offer their product based on an initial cost that they themselves suggest [18]. Thus, forest surplus owners in high-risk deforestation areas would likely suggest the highest CRA prices.

## Materials and methods

### Identifying governance requirements

To identify the main aspects needed to develop the CRA market, I analyzed the content of relevant existing laws and draft regulations at state and federal levels and carried out semi-structured interviews with fifteen key stakeholders from federal and state governments, civil society and the private sector who are involved in the design of the CRA regulations and market. In addition, I was a participant observer at four meetings on forest code implementation which included discussions of the CRA. These meetings involved 52 participants drawn from civil society along with government officials at the state and federal levels. The information gathered in these processes allowed me to assess the state and prospects of the environmental rural registry (CAR) implementation in Brazil, which is one of the major prerequisites to participate in for the CRA market.

### Scenarios of current forest surplus and deficit

The analyses in this study focus on two critical implementation aspects identified during the interviews and legal analysis: properties eligibility to the CRA market based on their land tenure status and the need for the State Environmental Agency to validate property information declared in the environmental rural registry (CAR).

First, I assessed three scenarios for estimating current forests surplus and debt eligible for the CRAs in Pará State, where the scenarios vary the eligibility of properties with different land tenure status. In the first scenario, only properties with land titles can participate in the market. In the second scenario, only titled properties can issue CRAs, but parcels without land title can use CRAs to compensate for illegal deforestation in Legal Reserve areas. In the third scenario, all properties (with or without land titles) could issue and use CRAs for compensation. I used ArcGIS software for all the spatial analyses using property level data as described in the following section and in S1 File.

#### Database for the scenarios analysis

The database of properties used in this study includes parcels with land titles from the Legal Land Program and the National Institute of Colonization and Agrarian Reform at federal level, as well as titles from Pará Land Institute (Iterpa) at state level. The study also uses data from the environmental rural registry (CAR), but since it did not specify land tenure status of parcels, I treated them as non-titled in the analysis.

There were several overlaps between parcels in the databases. Overlaps can arise for various reasons, such as differences in scale between the databases, the level of imprecision of the georreferencing method, issues related to conflict and land disputes, false ownership declaration, including false declarations over nonexistent properties. Where overlaps exist between parcel boundaries, I simulated the process that the state environmental agency will conduct to validate CAR data. The steps involved in validation will be described in the next sections. Following the validation simulation, the following types of properties were excluded:

1. All properties with more than 5% of overlap with Indigenous Territories; Conservation Units; areas of *quilombola* communities (a special type of private property granted for descendants of runaway slaves); military areas and Land Reform Settlements (large public areas formed by a group of parcels granted by the federal government to landless families).
2. All properties with more than 5% of overlap with each other.

This 5% threshold is the same used up to 2015 by the environmental agency in Pará to validate information in the environmental rural registry (CAR).

The scenarios analysis only included private parcels outside of protected areas, as there is no publicly available database of parcels inside these areas that could be eligible for the CRA market. I also excluded Land Reform Settlements from this first part of the analysis as they are currently ineligible for participating in the CRA market (see next section for further details).

Table 2 summarizes the final database used in this part of the analysis. The total area of titled and non-titled properties corresponds to 15% of Pará State territory. See S1 File for a detailed description of the database.

**Table 2:** Summary of non-overlapping parcels used in CRA analysis.

Category of analysis	Number of parcels	Total area (hectares)
Titled properties	4,552	2,584,123
Non titled parcels (Legal Land Program)	20,099	2,626,574
Environmental Rural Registry	39,113	14,032,471
Total	63,764	19,243,168

#### Estimating forest surplus and forest debt for the CRA market

After defining the database of properties to use in the analysis, the next step was to estimate the potential forest surplus for the CRAs and the forest debt per property, in accordance with the Forest Code rules. To determine these estimates, I used data on deforestation and forest cover up to 2013 from the Project for Monitoring Deforestation in the Legal Amazon (Prodes). I also used data on secondary forest (regeneration) up to 2012 from the Terra Class project. Both Prodes and Terra Class are projects from the National Institute for Space Research (INPE) in Brazil and are the official statistics for forest cover status in the Amazon biome. S1 File details the steps used in this analysis.

Drawing upon these data, I determined each property’s amount of deforested area and the forest surplus eligible for the compensation mechanism in accordance with the Forest Code. The rules to determine eligibility varied based on the property size and location, along with the property deforestation status by 1996 (since the legal reserve used to be 50% instead of 80% by then). For example, medium and large properties with pre-2008 forest debt need to have a minimum legal reserve of 50% (instead of 80%) if they are located in regions designated as in “expansion and consolidation” under the Pará Economic and Ecological Zoning. S1 File describes all decision rules used in this analysis.

I also identified a legal conflict between the forest code (a federal law) and state law in Pará with impact in the eligibility of forest surplus for the CRA market. This conflict applies only to properties located inside Environmental Protection Areas (EPAs), a type of protected area where private property is allowed. State law is stricter for EPAs than federal laws, so the forest surplus would be lower if state law prevails. However, since it remains unclear whether federal or state law will prevail, I considered both scenarios (i.e. dominance of federal or state law) for the forest surplus and debt estimates.

#### Limitations

The Forest Code requires that property holders permanently protect some areas with high conservation value, such as a buffer along rivers and mountaintops. These areas, known as Areas of Permanent Protection (APPs), are not eligible for the CRA mechanism. However, this study is unable to account for these areas, as publicly accessible geospatial information on the extent of these areas in Pará is lacking. Therefore, this study may overestimate forest surplus and debt.

In addition, I recognize that parcels excluded from the analysis due to overlap above 5% may eventually regularize their situation and have their CAR validated. In some cases, they could regularize their property by simply correcting their property boundaries. Thus, additional areas may become eligible to participate in the CRA market in the following years in Pará. However, correction of CAR data usually happens during validation. To date, the State Environmental Agency has been slow in validating CAR. Therefore, it is unlikely that many new parcels will become eligible in the years ahead beyond what I describe below.

### Five-year projection of the CRA market

Since some parcels are in the process of receiving land title and because land settlements may become eligible for issuing CRA in the next few years, I estimated a five-year projection of new CRA certificates entering in the market. I assumed that the forest deficit would remain the same as estimated in the first three scenarios of this study, as only legal reserve areas deforested before July 2008 can use the CRA to meet their forest code obligations. These five-year projections includes both private properties and land settlement projects.

#### Projection for CRAs in new titled-properties

Two different institutions preside over issuing titles at the federal and state level: the Legal Land Program at federal level and Pará Land Institute (Iterpa) at state level. For each institution, I calculated an estimate of how many new land titles they will likely issue per year between 2016–2019. First, I ran an OLS regression using data on the number of titles issued every year between 2009–2015 to estimate the number of titles they will likely issue in the future. This was run using the following model: Titleshat = β_0_+β_year_X, where Titleshat is the number of titles issued at year X by each institution, β_0_ is the intercept, and β_year_ is the slope, or the expected increment in the number of titles issued each year. However, β_year_ was not statistically significant (p>0.79), so one cannot really assert with statistical confidence if the number of new titles per year is linearly increasing every year or if the observed increase is due to chance, during the observed period. Thus, I decided to use the mean of titles issued by each institution between 2009–2015 as the annual estimate of new land titles between 2016–2019.

Based on this annual projection of new land titles, I estimated the forest surplus that would likely enter the CRA market from 2015–2019 under both federal and state jurisdiction. I included 2015 in the projection, as the dataset for this year was not available to calculate the exact forest surplus of the new titles. To estimate the future annual forest surplus, I used a bootstrapping procedure. The analysis drew upon a pool on 20,099 parcels registered via the Legal Land program and 20,802 parcels registered in CAR overlapping with state jurisdiction, both groups considered as untitled properties by 2014. I drew a sample, without replacement, equal to the number of estimated new titles at each year from 2015 to 2019, for each jurisdiction, and summed the forest surpluses of the sampled properties to obtain one bootstrap realization of the forest surplus, for each prediction year. I repeated this process for a total of 1,000 bootstrap replications for each year. Then, I calculated the mean forest surplus of all 1,000 bootstrap replications and used this value as an estimate of the amount of forest entering the market annually. I also estimated a 90% “percentile bootstrap confidence interval” for each annual forest surplus using the 5^th^ and the 95^th^ percentile of each year’s 1000 forest surplus bootstrap replications. The statistical analysis was performed in Stata.

It is worth noting that the Legal Land Program mandate for issuing land titles ends in 2017. As of August 2016, it was not clear if there would be a new law maintaining the program or if the National Institute of Colonization and Agrarian Reform (Incra) would become responsible for new land titling in federal jurisdiction areas. If the program is discontinued, it is plausible to assume a decrease in the annual titling performance in 2018–2019 until the new institution is fully operating in the new role, but this aspect was not considered in this analysis.

#### Projection for CRA*s* in land project settlements

To complete the five-year projection, I also included the estimated forest surplus from land settlement projects. Such settlements have a special land tenure status: the parcels granted to landless families belong to the government until the emancipation of the settlement occurs, which happens at least 10 years after the settlement creation. During this ten-year period, Incra is in charge of settlement management. In addition, according to the forest code, illegal deforestation in such settlements up to 2008 does not need to be restored or compensated. Therefore, the forest debts in settlements areas is not included in this analysis.

I estimated that part of these settlements would only be available for the CRA market starting in 2017, according to the following required steps:

1. Each family parcel in the settlements needs to have its own registration in the environmental rural registry. However, as of 2015 only the boundaries of the settlements had been registered. To make this parcel-by-parcel registration, Incra was seeking financial support from the Amazon Fund that supports several initiatives aiming at reducing deforestation and increasing forest conservation in the Brazilian Amazon. Nevertheless, project proposals from federal agencies to the Amazon Fund have taken on average ten months between submission and funds disbursement [21]. Thus, if Incra submitted a proposal in May 2015, funds would be available by August 2016. Based on that, I estimated Incra would take six more months to contract companies to work on the parcels’ registration and, finally, to start registering the parcels.
2. After this step, each settler would need to formalize an agreement with the environmental agency to regularize any noncompliance with the forest code. For instance, even if deforestation prior to July 2008 does not need to be restored or compensated, illegal deforestation after such date needs to be restored.
3. Contracts between families and Incra need to be modified to allow settlers to make direct CRA transactions. The current rules on how the families can use the parcels are very strict and only allow direct use of the land by the settlers.

Based on all these premises, I considered an optimistic estimate of having one fourth of the CRAs in settlements eligible for the market in Pará per year, starting in 2017. The steps planned by Incra do not prevent the institute from establishing partnerships with other institutions, such as universities and nongovernmental organization, to speed up this process of registering individual parcels in CAR. However, it is likely that this type of situation would not cover a significant number of settlements before 2017 to impact this study’s projection.

## Results

### Requirements for the CRA market

#### Environmental rural registry—CAR

Having validated cadasters in the environmental rural registry (CAR) is the main prerequisite to advancing the CRA market. If landholders do not include their properties in this register and if the environmental agency does not validate the registered CARs, then the forest code implementation—including the CRA market—will be impeded.

As of May 2016, 3.2 million parcels occupying 324.9 million hectares have been registered in CAR in Brazil [22]. This amount corresponds to 81.6% of the total estimated area to be registered in CAR [22]. Even though the law mandated that all rural properties register in CAR as of May 2016, in June 2016 the federal government promulgated a new law postponing this deadline to December 2017 with a possibility for further extension until December 2018 (Article 4^th^ of Federal Law number 13,295 from 2016). This postponement may delay the development of the CRA market, since many properties with a possible CRA demand could register in CAR later in 2018.

In addition, simply registering a property in CAR is not sufficient to make it eligible for the CRA market. The state environmental agency must check and validate all the information declared by the landholders in their registration. However, the validation phase to date has proven extremely lengthy. For instance, Pará started CAR registration in 2006, but only 2% of the CAR database used in this study had been validated by the State Environmental Agency as of October 2014 (2,762 registries out of 129,213.

Moreover, as of August 2016, the software used by most of the environmental agencies to validate CAR information (SICAR) did not have automatic settings to prevent some potential risks of inaccuracy or inconsistency data. For instance, even though the system informs the agency’s official about how complete their verification is (e.g., 50% of items have been verified), the official can in fact validate a CAR with low levels of verification. In practice, a CAR can be considered as validated even with very low percentage of items analyzed through SICAR, such as 5%.

Each state is responsible for setting its own regulations and controls to validate CAR data, which can include obligation to complete a minimum percentage of verification in each CAR before the agency considers a CAR as validated. Thus, depending on the differences among such state regulations, there will not be a unique parameter for CAR in Brazil, but different types of CAR per state, considering their level of reliability and control. The risk that varying the verification protocols poses to CRA market development is that some states may then possess lower quality environmental rural registries than others, which could result in the introduction of non-reliable CRAs in the market.

#### Land tenure

Another major variable influencing CRA market eligibility is the land tenure status. This is an important limitation in Pará because of the state’s problematic land tenure situation. Pará has been the leader in land conflict statistics in the Amazon over the last fifteen years. Estimates indicate that 39% of its territory lacks land tenure definition [23]. Solving this problem will involve issuing new land titles; validating and resolving conflicts from land titles issued in the past; and cancelling false land titles registered with notaries. However, the pace of agencies in pursuing all those activities has been slow.

Three major problems can account for why land regularization has been very slow. First, there is poor coordination among agencies in charge of land regularization at state and federal governments [24]. Even the actual boundaries of each jurisdiction are not entirely clear. In addition, state and federal governments dispute ownership over some areas. It is also common to have municipalities partially covered by state areas and by federal areas, so it would be desirable to have joint activities between state and federal agencies in order to solve pending situation at municipal level.

The second barrier to swift land regularization includes outdated procedures for analyzing land regularization claims, mainly at Iterpa (the state land agency). For example, Iterpa must check if a given parcel requesting a land title complies with environmental law before granting a land title, including maintenance of the required legal reserve. A trained agency official could assess this requirement with satellite images instead of making a field visit [24]. However, Iterpa continues to rely on long and expensive field visits to check legal requirements for granting land titles.

Finally, a third impediment to quicker land regularization is insufficient human resources [24]. Even if the land agency methodology of work is modernized and becomes less reliant on field visits, many current staff at these agencies are close to retirement age and will need to be replaced. At the same time, as replacements are found, the composition of skills and expertise among the staff would likely need to change in order to use new monitoring and implementation technology well.

Since 2011, the Legal Land Program has invested in software development and simplification of procedures to speed up the rate at which they respond to land claims assessments in federal jurisdiction areas [24]. The impact of such measures was only visible in 2014. For instance, in 2014 the program got the closest ever to reach its annual target of title issuance: 98% of the 10,673 titles target for all Amazon states [16]. Nevertheless, after the presidential impeachment in Brazil in 2016, staff both changed and were reduced, which further delayed the program.

At state level, Iterpa has recently announced that is working on a new system to increase the institution’s performance in landholding regularization [25]. However, given that this system is in initial phase of preparation, it is likely that the agency performance will remain the same for the next few years. The slow performance of land agencies in Pará will affect the potential supply of CRA from private properties in the short to medium term, as the next section explains.

Last, considering the current federal and state context, this analysis estimates that the number of new annual land titles in 2016–2019 will correspond to the mean number of titles issued in 2009–2015, which is 435 titles at State level and 666 titles at federal level per year.

### Current CRAs scenarios

**Fig 3:**
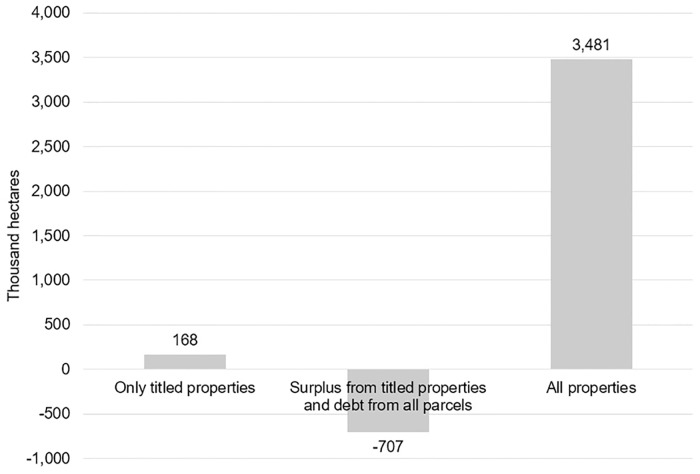
Sum of forest surplus minus sum of forest debt in three scenarios as of 2014.

Of the three current scenarios in this study, the total forest surplus for compensation exceed forest debt when only titled properties are allowed to participate in the market (scenario 1) and when all properties can issue and use the CRA for compensation (scenario 3) (Fig 3). These scenarios do not include potential CRAs in land reform settlements, since they currently do not fulfill conditions for engaging in this market, as discussed in the methodology.

If the government wants to use CRA as a way to promote low cost environmental regularization of properties, these results may indicate that the third scenario would be ideal for two reasons: i) all parcels could solve their environmental liability up to 2008 and ii) with a greater forest surplus than deficit, the value for the CRA would likely be low. However, the potential low value for CRA might not be enough to convince part of the landowners with forest surplus to engage in this market. This would be true especially in forest areas beyond 80% of each property where legal deforestation is still possible. The amount of such forest eligible for legal deforestation corresponds to 15 to 18% of the existing forest surplus (Table 3). In addition, incentives for forest restoration would be low under the third scenario, since all parcels could regularize deforestation up to 2008 through compensation instead of choosing to restore in situ.

**Table 3:** Description of forest debt, forest surplus and areas eligible for legal deforestation in three scenarios.

Scenario	Number of parcels	Forest debt (hectares)	Forest surplus (hectares)	Forest surplus eligible for legal deforestation (hectares)
1st: forest surplus and debt only from titled properties	4,522	228,958	396,671	71,165
2nd: forest surplus from titled properties and forest debt from all properties (including non-titled)	63,764	1,103,382	396,671	71,165
3rd: forest surplus and debt from all properties	63,764	1,103,382	4,584,816	670,632

Thus, if the government wants to use the CRA as an instrument to foster forest conservation in private properties and to stimulate restoration, restricting participation to sellers with title, while all can buy CRA certifications is potentially the most favorable scenario. The low forest surplus compared to forest debt could elevate the CRA value and is more likely to create real incentives to attract landowners with forest surpluses to participate in this market.

Finally, between 30% and 35% of the forest surplus in the three scenarios comes from forest areas undergoing the regeneration process (Table 4). Even though there is no legal difference between a CRA from primary or secondary forests, similar PES experiences in other countries have resulted in low payments for areas under regeneration compared to native forests [14]. Thus, lower CRA prices for regeneration areas may also become a possibility in Brazil.

**Table 4:** Proportion of forest regeneration in forest surplus for CRA scenarios.

Scenarios	Forest surplus (hectares)	Percentage of forest surplus from forest under regeneration
1st and 2nd	396,671	30
3rd	4,584,816	35

Last, the results assume that state law—which is more stringent—will prevail over federal law. However, if the federal law prevailed instead of state law, the estimates of forest surplus would increase between 3% and 24% and the forest debt would decrease from 1% to 2% across the three scenarios. S2 File presents the results of this legal conflict analysis.

### Five-year projection for the CRA market

**Fig 4:**
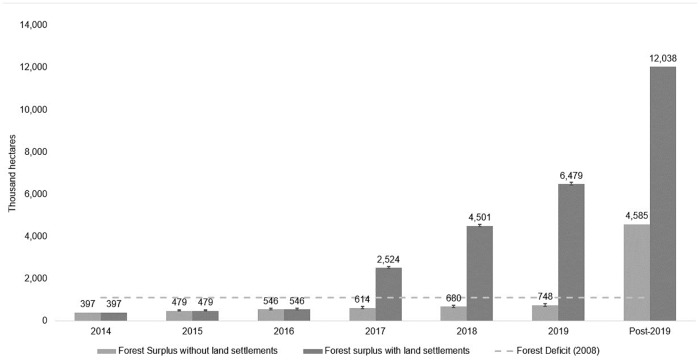
Estimated five-year projection (with the mean and 90% confidence interval) of forest surplus in Pará State with and without land settlements.

Over five years (2015–2019), the forest surplus eligible to participate in the CRA market could reach 68% (90% CI of 63% to 75%) of the estimated forest debt if land reform settlements do not enter the market: the forest surplus would rest at 746, hectares (90% CI of 693,108 ha to 828,163 ha) in 2019 compared with 1,103,382 hectares of forest debt. However, if settlements are permitted to issue, in three years the forest surplus would exceed debt in Pará: forest surplus would reach 2,524,000 hectares (90% CI of 2,486122 ha to 2,590,536 ha) in 2017 (Fig 4), which is more than double the (unchanged) estimated forest debt. S2 File presents the detailed projection results for each year.

Without including land settlements, the potential forest surplus for the CRA market will be insufficient to meet potential demand from forest debt. On the other hand, if land settlements become eligible to trade CRAs, they could flood the CRA market. This situation would likely result in low CRA prices, which in turn would reduce the incentive for owners of the forest assets to participate. The limited incentive to participate in the CRA market would be most prominent among landowners who face more remunerative alternatives, as we would expect to be the case in areas with high deforestation pressure. As will be discussed later, depending on the intended goal of the CRA program (conservation or low cost regularization), some regulatory options could help managing the amount of forest surplus for CRA so as to achieve the desired goals.

### Comparison with previous analyses

**Fig 5:**
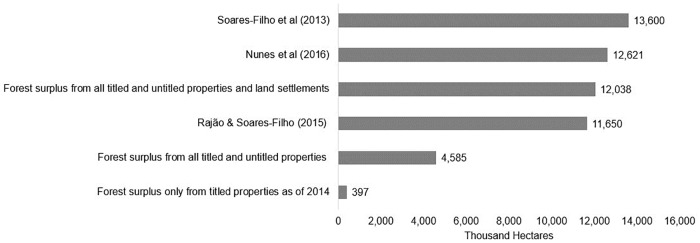
Comparison of CRA supply scenarios with previous studies.

The forest surplus estimates for CRA in Pará are 3–40% of the next smallest estimate from previous studies, except when adding land settlements. When including forest surplus from land settlements, the estimated forest surplus is 88–95% of the two highest estimates (Fig 5). Even though more recent studies removed cases without land titles from their estimate [11,12], their numbers are still 2.5 times larger than the highest estimate in this study without settlements. The authors from these studies explain that they likely overestimated the potential supply from titled properties due to uncertainty about the data on land tenure status (e.g. using non-titled parcels as titled properties) [11].

In addition, I argue that simulating validation in the environmental rural registry (CAR), which further reduces forest surplus estimates, is yet an important step to include when estimating properties eligible for the CRA market. Given that both overlap problems are frequent in the environmental rural register and that the agencies have been slow in detecting and resolving this issue, the number of cases that will qualify for CRA will likely be low in the next years. Thus, the scenarios proposed by previous studies could be considered as the potential CRA supply if all governance conditions to implement the CRA were met without incident. In contrast, estimates reported in this study represent forest surplus for CAR under existing governance conditions.

## Discussion

This study estimated a maximum forest surplus for CRA that is 2.5 less than the next closest estimate from previous studies, when excluding land settlements. This finding confirms the first study hypothesis that accounting for program implementation details and existing governance conditions would reduce the potential forest surplus for CRAs. In addition, the analysis showed how including land settlements could increase the forest surplus, such that forest surplus would exceed the estimated forest debt by a factor of 11. This links to the second hypothesis, in which I estimate that the total pre-2008 forest debt would only be fully compensated with CRA as of 2019 if land settlements become eligible to participate in CRA transactions.

Given the variation in potential outcomes among these scenarios, the Pará government’s choice of CRA implementation details—specifically the forest surplus eligibility rules and the supremacy of state over federal rules—will determine the extent to which this program serves as a tool to foster conservation or a way to lower the cost of environmental regularization of properties that had illegally deforested areas as of 2008. As recently as 2016, various key actors of the state government have been debating the goals of the CRA program. For instance, in a public event held in 2016 to discuss measures to decrease deforestation and enhance forest restoration in Pará, the State Secretary of Agriculture declared that the goal of CRA should be to allow a low cost regularization for rural properties. On the other hand, at the same event the Secretary for the Green Municipality Program (a state initiative to support environmental compliance at municipal level) stated a desire to find a balance between regularization and conservation with this mechanism.

If the government’s intention is to use CRA primarily as a way to lower the cost of environmental regularization of properties with illegal deforestation as of 2008, then the oversupply scenario will support such a goal. In this situation, there is no concern for restricting the market. However, if the government wants to use the CRA as a tool to foster conservation, the eligibility to supply CRA’s should be restricted. There are at least two possible ways to impose such limitation and encourage conservation.

First, the Pará state government could restrict CRA participation to the regions with the highest deforestation rates, under the assumption that those areas face the highest continued deforestation pressure. Via the Green Municipalities Program, Pará has already identified six municipality-based categories according to the degree of deforestation pressure and to municipal participation in the Green Municipalities Program [26]. The categories are as follows:

1. Embargoed: these municipalities have the highest rates of deforestation in the Amazon region and are featured in a black list from the Ministry of Environment;
2. Under pressure: these municipalities have high rates of annual deforestation, but are not yet featured in the black list;
3. Consolidated: these municipalities have medium risk of deforestation, less than 60% of forest cover and low annual rates of deforestation;
4. Forest base: for municipalities with high forest area and low risk of deforestation;
5. Green municipality: for those that were in the black list of the Ministry of Environment and now have low deforestation rates; or municipalities not included in the black list, but that comply with all environmental targets set by the Green Municipalities Program;
6. Nonparticipant: for municipalities not participating in the Green Municipalities Program.

To support conservation, the Pará government could, for example, only allow CRA to be issued in properties in the municipalities where deforestation is a greater threat. For example, limiting the eligible forest surplus for CRA to the municipalities in the “under pressure” category would result in a forest surplus of 1,090,084 hectares, including both titled and non-titled properties (Table 5). This would be 99% of the estimated potential forest debt. Table 5 presents the total forest surplus in each municipality category.

**Table 5:** Estimated forest surplus by tenure status and Green Municipality Program categories.

GMP category	Titled properties only (hectares)	Titled and non-titled properties (hectares)	Total with land Settlements (hectares)
Consolidated	56,269	528,736	1,129,452
Embargoed	90,276	1,411,044	3,227,624
Forest base	44,958	319,341	1,407,158
Green municipality	164,330	686,657	1,001,760
Nonparticipant	21,987	548,853	1,731,180
Under pressure	18,852	1,090,084	3,541,179
Total	396,671	4,584,715	12,038,352

A possible drawback to this approach is that landowners from non-selected zones could likely bring lawsuits against this stricter regulation, arguing that the deferral Forest Code did not impose such a restriction. However, the legal principle of specialization permits states to adopt regulations that are stricter than federal laws: the federal law is thus the minimum that needs to be complied with, not the maximum.

A second possible way to regulate the potential CRA supply is for the government to purchase the low cost CRAs issued from non-additional areas. In this situation, the goal would be to remove from the market the CRAs from areas that would be conserved in any case, such as properties located in consolidated and forest base zones. Some may argue that having the government as a buyer of CRAs is a waste of public funds, since such resources would not go into areas that need real incentives to prevent environmental damage. However, it is worth noting that by eliminating the cheap CRAs from the market, even if these CRAs are issued for a short duration, the government would be indirectly stimulating restoration of cleared areas whose owners could not find or buy CRAs. Therefore, conservation and restoration in those areas would receive a stimulus at a low cost by the government.

In order to maximize the amount of conservation achieved per dollar of governmental funds in pursuing this approach and also make price differentiation feasible and legal, governments could buy CRAs through reverse auctions. In this type of auction, landowners could offer their CRA based on an intended selling price, which could streamline government selection of the lowest-cost CRAs. The landowners could indicate their intended CRA price during the process to issue the CRA certificates in their properties. The Rio Green Stock Market (BV Rio) provides some precedent for up-front disclosure, as they already require property owners to declare their expected cost for selling or buying CRAs.

Finally, Pará could limit forest surplus by only permitting within-state transactions. As discussed previously, Pará currently permits interstate trade if the landowner with forest debts can prove that it is not economically and technically viable to compensate their debts with a forest surplus originated in Pará. However, particularly given the high potential of oversupply in the medium and long term, restricting the CRA transactions to within Pará would better align with goals of achieving conservation in areas that face higher deforestation pressures.

## Conclusion

Creating incentives to forest conservation in Brazil is an important step in maintaining the environmental gains of the last ten years, when deforestation in the Amazon dropped substantially. Such incentives are also critical if the Brazilian government intends to achieve an even greater reduction in deforestation rates in the future. The Forest Code passed in 2012 presents an opportunity to use the environmental reserve quotas as an instrument to support forest conservation on private properties. However, this study indicates that additional regulation may be necessary to make this instrument attractive for landowners in areas under high deforestation pressure in Pará.

The major methodological contribution of this study compared with previous literature is in how it accounts for governance conditions and implementation details when assessing the potential forest surplus and forest debt for Pará’s CRA market. More specifically, the two main governance features that distinguish this work are 1) varying the eligibility of properties to participate in the CRA based on land tenure status and 2) simulating the validation process of the environmental rural registry (CAR), which is a prerequisite of CRA participation.

By including these two critical implementation details, this study finds a maximum forest surplus for CRA 2.5 times less than the next closest estimate, excluding land settlements. In addition, the study demonstrates that permitting land settlements to issue CRA’s would likely flood the market and reduce both additionality in the market and the incentive to restore deforested areas. While these results are largely consistent with existing literature that projects greater potential forest surplus compared with potential forest debts, the main contribution of this article is by demonstrating that the oversupply scenario will likely not happen before three conditions are met: i) the land titling process for private landholdings advances and ii) the State environmental agency validates pending CAR submissions and iii) the Federal government decides on the eligibility of land settlements to supply CRA’s in the market. Until these activities happen, the forest debts in Pará will exceed the eligible forest surplus, and some landowners with forest debts will be required to restore deforested lands.

If the Pará government intends to use the CRA as conservation tool, a main recommendation from this study is to include additional criteria in the state legislation to limit the forest surplus eligible for CRA. In fact, this suggestion applies to all Brazilian states that have a potential forest surplus larger than the forest debt and that intend to use CRA as a tool to promote forest conservation on private properties. Such measure can increase the chance that the CRA mechanism supports additional conservation and reduce deforestation, especially in areas where deforestation is still legally permitted and the pressures are high.

## Supporting information

S1 File Detailed explanation on the database of rural properties and methods for determining forest surplus and debt per property. (PDF)

S2 File Additional results on forest surplus estimates. (PDF)
