# Supplementary material for: Potential trajectories of the upcoming forest trading mechanism in Pará State, Brazilian Amazon
Source: PLoS One. 2017 Apr 5;12(4):e0174154. doi: 10.1371/journal.pone.0174154 (PMC5381787; doi:10.1371/journal.pone.0174154)
Supplement: S1 File — (PDF) [file pone.0174154.s001.pdf]

S1 File

Potential Trajectories of the Upcoming Forest Trading Market in Pará State, Brazilian Amazon

Brenda Brito

Detailed explanation on the database of rural properties and methods for determining forest surplus and debt per property

Database of rural properties

This study used a database to estimate potential forest surplus and debt eligible for CRA in Pará with the following information: parcels with land titles issued by the federal and state land agencies (Incra and Iterpa); areas of *quilombola* communities – a special type of private property granted for descendants of runaway slaves; georeferenced parcels undergoing the tenure regularization process, in addition to properties signed up to the Environmental Rural Registry of Pará (Table A). All analyses were performed using the Lambert Conformal Conic projection and Sirgas 2000 Datum.

Table A. Database used in this study

**Geographic database**	**Source**	**Year**
Certified properties	Legal Land Program	Unspecified
Deforestation	Prodes/Inpe	1997-2013
Economic Ecological Zoning of Pará	Pará state environmental agency	2014
Federal and State Protected Areas	Instituto Socioambiental (ISA)	2010

Federal Land Reform Settlements	Incra	2013
Forest	Prodes/Inpe	2013
Non-Titled parcels	Legal Land Program	2013
Hydrography	Prodes/Inpe	2012
Indigenous lands	Funai	2010
Parcels in the Environmental Rural Registry pending validation	Pará state environmental agency	October 2014
Secondary vegetation	Terra Class/Inpe	2012
Titled properties by Incra	Legal Land Program	Unspecified
Titled properties by Iterpa	Iterpa	2011
Titles properties by the Legal Land Program	Legal Land Program	2013
Quilombola territories*	Incra(SR01), Iterpa	2011
Vegetation typology	Prodes/Inpe	2012
Parcels in the Environmental Rural Registry validated by the state environmental agency	Pará state environmental agency	October 2014

* Refers to territories from runaway slave descendants' communities (*Quilombolas* in Portuguese)

First, I eliminated from the analysis private properties overlapping with protected areas, military areas and land reform settlements using a 5% overlap threshold. Thus, I excluded all overlapping parcels above this limit. To calculate the overlaps in this study, I used the Tabulate Intersection tool of ArcGIS software.

Then, I divided the analysis into three categories of properties: titled properties, non-titled parcels, and parcels in the Environmental Rural registry. The titled properties category includes land titles issued by the Ministry of Agrarian Development between 2009-2013 through a program called Legal Land; properties whose georeferenced maps were certified by the Federal Land Agency (Incra); land titles issued by the State Land Agency (Iterpa) and land titles issued by Incra. I eliminated parcels with more than 5% overlap within this first category.

The second category (occupations) includes parcels georeferenced by the Legal Land Program (from the federal government) up to August 2014. These parcels are in the process of obtaining a land title from the Legal Land Program. I excluded non-titled parcels overlapping more than 5% with the first category (titled properties) and within this second category. Finally, the third category (parcels in the Environmental Rural registry) includes parcels whose CAR information was validated by the environmental agency and those pending validation. I eliminated: i) parcels in CAR with more than 5% overlaps from the previous two categories (titled and non-titled category); ii) overlaps above 5% within CAR parcels (pending and verified), and iii) overlaps above 5% between pending and verified environmental registry, prioritizing validated CAR over pending validation. Table B summarizes the steps for eliminating overlaps.

Table B. Summary of criteria to eliminate overlaps among parcels

Type of parcels in which criteria to eliminate overlaps was applied	Eliminate 5% overlap with:
All parcels: titled properties, non-titled parcels and parcels in the Environmental Rural Registry (CAR)	Indigenous Lands, Protected Areas, Military area, Land Reform Settlements
Non-titled parcels and parcels in the Environmental Rural Registry (CAR)	Titled properties
Parcels in the Environmental Rural Registry (CAR)	Non-titled parcels
Parcels in the Environmental Rural Registry (CAR) pending validation	Validated Enviromental Rural registry

After that, I excluded properties with more than 99% overlap with water bodies and non-forest vegetation and calculated the area of non-forest vegetation on the remaining properties. I then used the resulting properties as the basis for applying the Forest Code rules for identifying forest surplus and debt up to July 2008. For example, if the law requires that the property must have 80% of forest vegetation as Legal Reserve, this percentage was applied to the area of the property excluding the non-forest vegetation. Table C presents the final dataset used in this analysis.

Table C. Final database after eliminating overlaps above 5%

**Category of analysis**	**Type of parcel**	**Number of parcels**	**Total area (hectares)**
Titled properties	Titled parcels by Iterpa	91	246,317
Titled properties	Certified properties	401	1,677,745
Titled properties	Titled parcels by Incra	2,188	135,361
Titled properties	Titles issued by the Legal Land Program	1,845	147,176
Titled properties	Quilombola territories	27	377,524
Non titled parcels	Georeferenced parcels	20,099	2,626,574
Environmental Rural Registry	Verified Environmental Rural Registry	1,816	3,704,234
Environmental Rural Registry	Pending Environmental Rural Registry	37,297	10,328,237
	Total	63,764	19,243,168

Method for determining forest surplus and debt per property

To determine forest surplus eligible for CRA, I used deforestation data from the Prodes database up to 2013 and secondary vegetation according to the Terra Class database up to 2012. I excluded from the Prodes database the overlapping areas of secondary vegetation in Terra Class because Prodes does not identify which deforested areas are in the process of regeneration. Then, I used this new database of *Prodes without secondary vegetation* to calculate deforested areas up to 2008 that could be eligible for compensation through CRA. Any deforestation after 2008 must be restored in situ and cannot be compensated elsewhere according to the forest code.

To estimate the forest surplus in Pará coming from primary and secondary forests, I followed three steps:

i. Using data from Prodes 2013, I excluded the non-forest vegetation area and the deforested area on each property. The result was the remaining primary forest on each property. Next, I calculated if each property had the minimum percentage of legal reserve and the amount of forest eligible for CRA. The legal reserve percentage varied according to size and location of area (see Table D for more details)

ii. Using data from the new layer *Prodes without secondary vegetation*, I excluded from each property the non-forest vegetation areas and the deforested areas. The result was the sum of the existing primary and secondary forest. I then calculated whether each parcel had the minimum legal reserve and the amount of forest eligible for CRA.

iii. I subtracted the step two output from step one results to determine the forest eligible for CRA formed only by secondary forests.

In general, the forest code requires a legal reserve of 80% allows the issuance of CRA certificates for forest areas above this percentage. However, there are several exceptions to this rule. In some cases, all existing forest cover on the property are eligible for CRA; in other cases, only forests above 50% of the property can be transformed into CRA. For deforested areas eligible for compensation, exceptions also apply, so the percentage can vary between zero to eighty. To determine which rule to use, I considered the following factors according to the forest code:

i. Size of the parcel in fiscal module: a unit used by the government to measure rural properties. A fiscal module average is 64 hectares in Pará State. In this analysis, I calculated the number of fiscal modules per property and, for properties located in more than one municipality, I considered the average fiscal module size.

ii. Location of property: in some circumstances, the legal reserve is reduced from 80% to 50% only for the purpose of regularization. However, this specific rule is not a permission to deforest legal reserve above 50%: if there is forest between 50-80%, it is considered legal reserve and new clearings are not allowed. No matter the size or location of the property, the Forest Code permits new deforestation only in the forest exceeding 80% of the property.

If the legal reserve was deforested as of 2008, the property owner needs to regularize it up to 50% instead of 80%. This specific rule applies in the following

cases: i) for properties in municipalities located in areas designated as in expansion and consolidation according to Pará Economic and Ecological Zoning or ii) when the municipality has more than 50% of its territory as protected area (Indigenous lands and conservation units).

iii. Existence of 50% of forest cover by 1997: up to1996, the Legal Reserve area in the Amazon forest region was 50% of the property. Those who had complied with the 50% rule by 1996 can follow this limit to issue or use CRA for compensation. However, to apply this rule in this analysis, I used accumulated deforestation data up to 1997, since this is the oldest disaggregated data provided by Prodes. Moreover, I identified a legal conflict when applying this rule to properties inside environmental protection areas (EPA), a type of protected area that allows private property. A state law that establishes the Ecological Economic Zoning of Pará considers that the legal reserve percentage of properties inside EPAs is 80% (regardless of whether or not there was 50% forest cover by 1996). Thus, I considered both scenarios (prevalence of forest law and prevalence of state law) for estimating CRA supply and demand in properties inside EPAs.

Table D summarizes the rules used to calculate the forest surplus and debt eligible for using CRA.

Table D. Summary of rules for determining forest surplus and deficit eligible for CRA

Size (fiscal module)	Location of property			Maintained 50% of forest cover in 1997	Forest surplus eligible for CRA	Area deforested up to July 2008 eligible for compensation (legal reserve debt)
	Areas designated as in expansion or consolidation under the Pará Economic and Ecological Zoning	Municipality with more than 50% of its size as protected areas	Inside enviromental protection areas			

Up to 4	Does not matter	Does not matter	No	Does not matter	All existing forest cover	None
Over 4	Yes	No	No	Does not matter	Forest > 50% of property	Up to 50% of property
Over 4	No	Yes	No	Does not matter	Forest > 50% of property	None
Over 4	No	No	No	Yes	Forest > 50% of property	Up to 50% of property
Over 4	No	No	Yes	Yes	Conflict between forest code and state law, thus two possible scenarios: Forest > 80% of property or Forest > 50% of property	Conflict between forest code and state law, thus two possible scenarios: up to 50% of property or up to 80% of property
Over 4	No	No	Yes	No	Forest > 80% of property	Up to 80% of property
Over 4	No	No	No	No	Forest > 80% of property	Up to 80% of property
