# Supplementary material for: Potential trajectories of the upcoming forest trading mechanism in Pará State, Brazilian Amazon
Source: PLoS One. 2017 Apr 5;12(4):e0174154. doi: 10.1371/journal.pone.0174154 (PMC5381787; doi:10.1371/journal.pone.0174154)
Supplement: S2 File — (PDF) [file pone.0174154.s002.pdf]

S2 File

Potential Trajectories of the Upcoming Forest Trading Market in Pará State, Brazilian Amazon

Brenda Brito

Additional results on forest surplus estimates

Impact of the legal conflict between state law and forest code in the estimated forest surplus eligible for the CRA market in Pará State

When the federal forest code rules prevailed over state rules, the estimated potential forest surplus increased between 3 and 24% (Table A) from the scenarios estimated in the paper. The key difference between the federal and state laws is that the federal law allows a legal reserve of 50% in environmental protection areas (EPA), if this same percentage was conserved by 1996, while the state law requires legal reserve of 80% of the property located in EPA, irrespective of conservation baseline year.

Thus, the federal law would permit forest areas in EPA above 50% of the parcel to issue CRAS (given they conserved by 1996), whereas the state law would consider only lands in excess of the 80% requirement to be eligible to issue CRAs. At the same time, the area of forest debt would also be 1 to 2% smaller if the federal forest code prevails, since compensation would be up to 50% instead of 80%, according to the abovementioned condition.

The state of Pará could resolve his conflict by invoking the principle of specialization, whereby a more specialized law (generally a state level law) can prevail over a more general law (usually

a federal law). In this case, the more specialized, and stricter state level laws would prevail, and the federal law could be interpreted as setting a general minimum, for states to comply with. Table A demonstrates the effect of invoking specialization or deferring to the more general federal forest code.

Table A. Compensation balance in three scenarios and variation between precedence of State and Federal laws

Scenario	State law prevails		Forest Code prevails	
	Forest debt (hectares)	Forest surplus (hectares)	Forest debt (hectares)	Forest surplus (hectares)
1st: forest surplus and debt only from titled properties	228,958	396,671	224,165	406,445
2nd: forest surplus from titled properties and forest debt from all properties (including non-titled)	1,103,382	396,671	867,474	406,445
3rd: forest surplus and debt from all properties	1,103,382	4,584,715	1,091,638	4,698,840

Estimated projection of forest surplus per year

Table B. Descriptive statistics for the projection of forest surplus considering new land titles in 2015-2019.

Forest surplus		2015	2016	2017	2018	2019
Without land settlements	Mean	478,571	546,303	613,743	680,461	747,651
	5th Percentile	461,476	517,165	575,710	634,101	693,108
	95th Percentile	527,119	608,255	680,124	755,117	828,163
With land settlements	Mean	527,119	608,255	2,524,155	4,501,284	6,478,886
	5th Percentile	461,476	517,165	2,486,122	4,454,924	6,424,343
	95th Percentile	527.119	608.255	2.590.536	4.575.940	6.559.398
Standard deviation from non-titled properties		20.456	28.839	34.486	39.183	44.570
